# Remote Design of a Smartphone and Wearable Detected Atrial Arrhythmia in Older Adults Case Finding Study: Smart in OAC – AFNET 9

**DOI:** 10.3389/fcvm.2022.839202

**Published:** 2022-03-21

**Authors:** Larissa Fabritz, D. Connolly, E. Czarnecki, D. Dudek, A. Zlahoda-Huzior, E. Guasch, D. Haase, T. Huebner, K. Jolly, P. Kirchhof, Ulrich Schotten, Antonia Zapf, Renate B. Schnabel

**Affiliations:** ^1^University Center of Cardiovascular Science, University Medical Center Hamburg-Eppendorf, Hamburg, Germany; ^2^Department of Cardiology, University Heart and Vascular Center Hamburg, University Medical Center Hamburg-Eppendorf, Hamburg, Germany; ^3^German Center for Cardiovascular Research, Partner Site Hamburg/Luebeck/Kiel, Berlin, Germany; ^4^Institute of Cardiovascular Sciences, University of Birmingham, Birmingham, United Kingdom; ^5^Department of Cardiology and R&D, Birmingham City Hospital, Sandwell and West Birmingham Trust, Birmingham, United Kingdom; ^6^Atrial Fibrillation NETwork, Münster, Germany; ^7^Institute of Cardiology, Jagiellonian University Medical College, Krakow, Poland; ^8^Maria Cecilia Hospital, GVM Care & Research, Ravennna, Italy; ^9^Department of Measurement and Electronics, AGH University of Science and Technology, Krakow, Poland; ^10^Institut Clínic Cardio-Vascular, Hospital Clínic, University of Barcelona, Barcelona, Spain; ^11^August Pi i Sunyer Biomedical Research Institute, Barcelona, Spain; ^12^Centro de Investigación Biomédica en Red en Enfermedades Cardiovasculares, Madrid, Spain; ^13^Preventicus GmbH, Jena, Germany; ^14^Institute of Applied Health Research, University of Birmingham, Birmingham, United Kingdom; ^15^Department of Physiology, Cardiovascular Research Institute Maastricht, Maastricht University Medical Center, Maastricht, Netherlands; ^16^Institute of Medical Biometry and Epidemiology, University Medical Center Hamburg-Eppendorf, Hamburg, Germany

**Keywords:** atrial fibrillation, screening, wearable, digital consent, stroke, telemedicine, digital cardiology, photo plethysmography

## Abstract

**Introduction:**

Screening for atrial fibrillation and timely initiation of oral anticoagulation, rhythm management, and treatment of concomitant cardiovascular conditions can improve outcomes in high-risk populations. Whether wearables can facilitate screening in older adults is not known.

**Methods and Analyses:**

The multicenter, international, investigator-initiated, single-arm case-finding Smartphone and wearable detected atrial arrhythmia in older adults case finding study (Smart in OAC – AFNET 9) evaluates the diagnostic yield of a validated, cloud-based analysis algorithm detecting atrial arrhythmias via a signal acquired by a smartphone-coupled wristband monitoring system in older adults. Unselected participants aged ≥65 years without known atrial fibrillation and not receiving oral anticoagulation are enrolled in three European countries. Participants undergo continuous pulse monitoring using a wristband with a photo plethysmography (PPG) sensor and a telecare analytic service. Participants with PPG-detected atrial arrhythmias will be offered ECG loop monitoring. The study has a virtual design with digital consent and teleconsultations, whilst including hybrid solutions. Primary outcome is the proportion of older adults with newly detected atrial arrhythmias (NCT04579159).

**Discussion:**

Smart in OAC – AFNET 9 will provide information on wearable-based screening for PPG-detected atrial arrhythmias in Europe and provide an estimate of the prevalence of atrial arrhythmias in an unselected population of older adults.

## Introduction

Atrial fibrillation (AF) is often only diagnosed in the context of a first stroke [up to 10% of unselected stroke survivors (1)]. Earlier initiation of anticoagulation could prevent strokes and systemic embolism, and reduce cardiovascular mortality in patients (2).

Recent controlled clinical trials demonstrate that population-based screening for AF and subsequent initiation of oral anticoagulation can reduce stroke in elderly populations (3, 4). These trials also illustrate a relatively high number needed to screen and that a relevant proportion of those invited to screening do not use patient-operated ECGs (3) or implanted monitors (4). Thus, simpler methods to screen for AF are desirable. Many consumer devices, most notably smartphones and smartwatch/wearable-based devices (5–7), enable near-continuous heart rhythm monitoring with reasonable precision (8–10). Such technologies could offer an additional, potentially simpler way of screening for atrial arrhythmias. So far, these promising technologies have mainly been evaluated in younger, tech-savvy early adopters (5), while the biggest clinical need for AF screening is in unselected elderly populations (11). Older adults, however, may face barriers in uptake, use and adherence to smartphone and app based screening offers. To advance the use of consumer electronics for AF screening, there is a need to evaluate the uptake, usability, and diagnostic yield of atrial arrhythmias in older adults with and without prior knowledge of wearable technologies.

The Smartphone and wearable detected atrial arrhythmia in Older Adults Case finding study (Smart in OAC – AFNET 9) will therefore evaluate the usability of a fully digital PPG-based detection system for atrial arrhythmias in an unselected population of older adults. The study will furthermore evaluate communication channels designed to offer PPG-based arrhythmia screening to older adults. This case finding study will also fully adhere to European privacy regulations.

## Methods and Analysis

### Study Design

**FIGURE 1:**
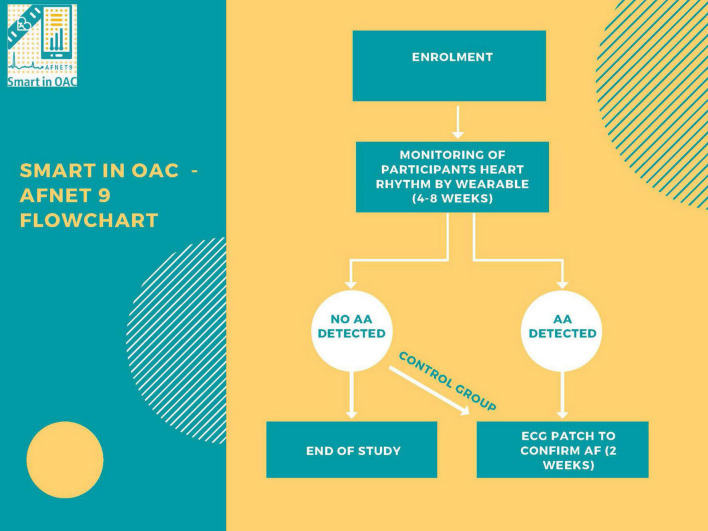
Study flow chart.

Smart in OAC – AFNET 9 is an investigator-initiated, single-arm, international, multicentre case-finding study in an at-risk population without previously known atrial fibrillation using a low-threshold, digitally enhanced screening platform (Figure 1). The primary objective of Smart in OAC – AFNET 9 is to determine the ability of a wearable-based PPG-based screening to detect atrial arrhythmias in older adults. Within the limitations of a controlled trial requiring consent, the system is designed for simplicity (Figure 1). We will estimate the detection rate of atrial arrhythmias using a validated PPG analysis system using a consumer electronic wearable in a structured, digital screening process offered to individuals aged 65 years or older. The study has been approved by the local Ethics Committees in all participating sites.

### Participants

Smart in OAC – AFNET 9 aims to reach out to unselected participants aged 65 years or older without previously known AF and not on oral anticoagulation and can provide informed consent. During enrolment, we rely on self-reported information provided by the participants. Several communication channels are used to reach the target population. It is planned to advertise the study using newspaper adverts and television, media that are commonly used by older adults. Study centres will additionally explore ways of approaching participants directly, suitable to the region’s cultural and regulatory peculiarities, e.g., those seen during routine health checks including vaccination appointments. The routes of contact will be described and evaluated during the study aiming to identify routes that enable equitable access to the screening tool.

### Study Intervention

Participants will be offered participation in the study using an online consent form. Paper versions are available if required by local ethics regulations. Participants who consent to the study will be provided with a wristband (Corsano 287, MMT SA, Switzerland) coupled to an existing smartphone (operating system requirements Apple iOS version 12.2 or higher or Android 8.0 or higher). The wristband contains a PPG sensor that couples to the participant’s smartphone via Bluetooth. Initiation of the study requires installation of the Corsano Preventicus Smart app on the smartphone. The PPG is recorded from the wristband automatically after pairing the wristband to the smartphone. Data is first stored in the wristband and automatically uploaded via smartphone to Preventicus Telecare^®^ cloud. The first pairing activation can be done by the participants themselves following in-app guidance. In-person support is provided via the local study teams and by trained staff at Preventicus. Once the monitoring is activated and the wristband coupled to the smartphone, the system enables continuous monitoring of heart rhythm using a wristband with a PPG sensor coupled with a smartphone app and a validated cloud based analytic service [Preventicus Heartbeats^®^, Jena, German^1^ (9)]. The wearable technology records passively around the clock, operating for up to 5 days without re-charging, and determines the length of atrial arrhythmia (AA) episodes and the AA burden per day using a fully automated, cloud-based transmission and analysis service. Participants are asked to wear the wristband and use the system for 4 weeks, including nights, with a possibility to extend monitoring up to 8 weeks. This screening duration was chosen by the steering committee as likely to be biologically meaningful in terms of stroke risk, as very rare atrial arrhythmias detected by long screening durations will be associated with a lower stroke risk than atrial arrhythmias detected during one to two months of screening (11–13).

All signals will be centrally analysed by the Preventicus Telecare service using Preventicus Heartbeats^®^. Preventicus is ISO 13485 certified. Preventicus Heartbeats^®^ is a cloud-based and device-agnostic analytic service for plethysmographic (PPG), accelerometric (ACC), and ECG raw data. It is a CE class IIa certified medical product for heart rate and rhythm analysis based on either 1.5 min measurements with smartphone camera, or continuous and passive raw PPG recordings from wearables. More than 10 million analyses have been performed so far with the analytic service; it has been comprehensively validated (14–17). The algorithm showed an accuracy of ∼96% and a positive predictive value of ∼99% for AF in the “WatchAF” and “Detect AF Pro” trials with more than 1200 participants (9, 18). To quantify which arrhythmias will be classified as atrial arrhythmia in the study, ECG beat annotations containing over 150,000 min of rhythm recordings from 341 subjects available in seven open databases available from PhysioNet were used (see Supplementary Table 1) to obtain 1-minute-long beat-to-beat segments that were evaluated using the AF-detection algorithm used in the study. The minute-wise comparison of ground truth arrhythmias and AA detection by the algorithm demonstrates good sensitivity and specificity for AF (see Supplementary Table 2). These analyses were furthermore compiled to provide a subject-wise picture of the arrhythmias contained. This evaluation demonstrates that the algorithm comprises atrial fibrillation. In addition to PPG-detected atrial arrhythmias, the investigational product also uses raw PPG data to detect other abnormalities with irregular beat-to-beat intervals and accurately differentiates them against sinus rhythm and AA (19).

When an atrial arrhythmia episode is detected and verified by the Preventicus Telecare service, participants are offered a 14-day external loop recorder Holter ECG (CardioMem^®^ CM 100 XT) (Figure 1). If the participant agrees to receive the ECG device remotely, the necessary equipment and instructions for use at home will be mailed. The study sites will provide the ECG loop device to the participant and collect additional health data. Participants will be informed about the results of the measurements and about whether further actions are to be taken depending on their results. Participants with clinically confirmed atrial fibrillation will be offered a clinical visit at the study site to determine their best management.

### Data Collection

Smart in OAC – AFNET 9 will collect all data remotely via the consumer electronic device enhanced with the smart wristband. During the enrolment, the participants provide information on data such as name, mobile number, date of birth, known AF and current oral anticoagulation and there is an option to fill in an EQ-5D-5L electronically (Table 1). A participant ID is assigned automatically. In participants with atrial arrhythmias detected by the PPG, additional information on cardiovascular conditions will be requested and further data such as repeat information on quality of life based on the EQ-5D-5L will be collected.

**TABLE 1:** Primary outcome and key secondary outcomes of Smart in OAC – AFNET 9.

**1. Primary Outcome** The primary endpoint is the proportion of participants with newly detected atrial arrhythmias within 4 weeks of device use of all participants included in the study. It will be reported with a two-sided 95% Clopper-Pearson confidence interval.
**2. Key Secondary Outcomes** Proportion of participants with atrial arrhythmias detected at any time, including those with atrial arrhythmias detected within the full time of recording will be reported with a two-sided 95% Clopper-Pearson confidence interval Time from completed enrolment to the first positive screening, taking death as competing risk into account will be analysed using Aalen–Johansen curves. Regional differences of atrial arrhythmia prevalence (diagnostic yield), differences by route of invitation and enrolment will be compared using a logistic regression model Differences by route of invitation and enrolment will be compared using a logistic regression model Compliance: The compliance of participants with protocol with regards to the measurement procedure of the app and wearable will be presented descriptively. This will include reasons to discontinue the monitoring prematurely, and reasons for withdrawal of consent. The duration of screening per participant will be plotted. Proportion of participants with atrial arrhythmias contacting the study centre (personal visit or remote),as recommended. Number of participants wearing the 14 day Tele ECG patch after detection of atrial arrhythmias; Compliance of participants using the app/wearable: percentage of active users after two weeks, histogram of analysable data recorded. Detection of AF: Number of participants with clinically confirmed arrhythmias (sub-analysis: AF) during Holter ECG, documented clinically or by event-recorder. We will add clinical evidence as available. The agreement of ECG-based detection of AF will be accessed by cross-tabulating both methods and quantified using the proportion of concordant diagnoses in the ECG subpopulation.

As technical difficulties could impair enrolment and adherence for the oldest participants, we plan technical support in local languages not only at inclusion, but also during follow-up to address any potential pairing and software issues. The app’s design for this study was specifically designed and adapted for ease of use, including enhanced contrast and enlarged font size for improved use in older adults.

Adherence to the screening programme and duration of PPG monitoring will be recorded as secondary outcome. If participants discontinue participation, the information gathered until discontinuation will be analysed.

Due to the device-agnostic certification, the Preventicus analysis service can be coupled with different wearables, provided they transmit PPG and ACC raw data (and optionally ECG data) to the cloud service in a standardised way. In the present study, the Corsano 287 wearable PPG wristband is used for this purpose. It is manufactured and provided by MMT SA (Switzerland^2^), an ISO 13485 certified medical product manufacturer. The core module of Corsano 287 has been previously used with over 150,000 modules sold and has CE medical device certification under EU-MDR standards. The wearables provide an up to 5 days recharge cycle and are suitable for continuous PPG and ACC raw data capturing including transfer to cloud service via Bluetooth 5.0 using the participants smartphone.

Data and information technology safety and data security requirements are met. Preventicus data management and data protection comply with General Data Protection Regulation. Personal data (declarations of consent, contact information, etc.) are stored exclusively in Preventicus Caresafe (i.e., a platform for the study centres to manage the digital enrolment modalities. The data in the Caresafe are end-to-end encrypted, so that Preventicus and Corsano Health B.V. (manufacturer of the app) and MMT (manufacturer of the wearable) are not able to gain access to personal data.

### Sample Size and Statistical Analyses

The prevalence of AA in elderly populations was ca. 30–40% when continuous monitoring is applied for 2–3 years using implantable loop recorders (4, 20, 21). Integrating the estimated effects of shorter monitoring times (1 month), considering that the wearable will not be used 24/7 by all participants, and based on the known effects of intermittent and shorter ECG monitoring on detection rates of short AA (7, 22), we estimate a detection rate of AA of 3–6% in the screening population. This estimated rate is higher than observed in STROKESTOP, where only very short intermittent monitoring was applied (30 s twice a day for a few weeks) (23).

A sample size of 1,000 participants undergoing PPG screening will allow us to estimate a rate of detection of 5% with a precision of 2.8% (width of the two-sided 95% Clopper Pearson confidence interval, PASS 16.0.3). For 750 participants, the precision is 3.3%, for 500 participants 4%.

Details will be set out in a statistical analysis plan. The primary analysis will be based on the full analysis data set, consisting of all participants that consented to screening and provided at least one data point. For the analysis of the demographics and baseline characteristics, descriptive statistics will be used. The proportion of participants that consent to participate in screening or not will be estimated with corresponding two-sided 95% Wald confidence interval. The detection rate of AA will be calculated together with the corresponding two-sided 95%-confidence interval.

### Primary Outcome

The primary outcome parameter of this study is the prevalence of PPG-detected atrial arrhythmias calculated as number of participants with AA detected by the wearable in relation to all included participants.

### Secondary Outcomes

Secondary outcomes include a description of the enrolment routes and comparison of clinical characteristics between enrolment routes, proportion of participants who underwent monitoring as a proportion of the participant invited, and the duration of monitoring per participant. Regional differences of AA prevalence within the European study sites in terms of AA prevalence and differences by route of invitation and enrolment will be evaluated. All processes and procedures will be evaluated to extract information on usability, including exclusions due to lack of smartphone ownership or digital capability. Further information collected for key secondary analyses is provided in Table 1.

### Adverse Events

This observational study uses approved technologies based on tested consumer electronics in an approved indication, evaluating the feasibility of its use to screen an at-risk population at large scale in a low-threshold access setting. Thus, Smart in OAC – AFNET 9 is a low-risk study. Adverse events related to the study procedures (e.g., side effects of the wearable, in this case a wristband) will be prospectively collected and reported.

## Discussion

Smart in OAC – AFNET 9 will provide information on the usability and diagnostic yield of screening for atrial arrhythmias in unselected older adults. The results will address the open question whether structured AF screening programmes using short-term ECG recordings or implanted rhythm monitors can be supplemented or replaced by consumer-electronic based arrhythmia screening. While a growing majority of older adults in the UK (24) and in Germany (25) now use a smartphone, the feasibility of the study part will also assess the extent to which digital exclusion might limit access to this technology in older adults.

The results will provide robust information on the prevalence of PPG-detected arrhythmias in older adults. Smart in OAC – AFNET 9 will evaluate and validate pathways for participant recruitment and follow-up and thus generate robust information for the planning of an outcome trial. Thereby, the study will provide data on different methods to reach out to such populations to offer arrhythmia screening and on characteristics of participants with PPG-detected arrhythmias.

## Ethics Statement

Smart in OAC – AFNET 9 is registered at https://clinicaltrials.gov/ct2/show/NCT04579159. Ethics have been granted (Hamburg 2020-10260-BO-ff, Dresden (Markkleeberg) EK-BR-95/21-1, Barcelona HCB/2021/0255, Krakow/Nowy Sasz 298/KBL/OIL/2020, Birmingham, UK IRAS 292218). The protocol of the study is published with this manuscript. The results of the trial will be published after trial completion and statistical analysis. A list of secondary analyses and publications will be provided by the steering committee of the trial based on the statistical analysis plan. The steering committee will be responsible for decisions regarding additional analyses and access to data.

## Author Contributions

LF, RS, and AZ wrote the manuscript. RS, PK, LF, AZ-H, DH, EC, and KJ designed the protocol. AZ, DD, EG, LF, TH, US, and RS planned the study. KJ, EC, AZ-H, LF, RS, and EG designed the patient-friendly information and prepared ethics applications. All authors made critical comments on the manuscript.

## Author Disclaimer

The views expressed are those of the author(s) and not necessarily those of the NHS, the NIHR or the Department of Health and Social Care.

## Conflict of Interest

LF has received institutional research grants and non-financial support from European Union, British Heart Foundation, Medical Research Council (United Kingdom), several biomedical companies and previously DFG. The Institute of Cardiovascular Research, University of Birmingham, has received an Accelerator Award by the British Heart Foundation AA/18/2/34218. LF and PK are listed as inventor of two patents held by University of Birmingham (Atrial Fibrillation Therapy WO 2015140571, Markers for Atrial Fibrillation WO 2016012783). US received consultancy fees or honoraria from Università della Svizzera Italiana (USI, Switzerland), Roche Diagnostics (Switzerland), EP Solutions Inc. (Switzerland), Johnson & Johnson Medical Limited, (United Kingdom). US is co-founder and shareholder of YourRhythmics BV, a spin-off company of the University Maastricht. RS has received speaker fees from BMS/Pfizer. PK receives research support for basic, translational, and clinical research projects from European Union, British Heart Foundation, Leducq Foundation, Medical Research Council (United Kingdom), and German Centre for Cardiovascular Research, from several drug and device companies active in atrial fibrillation, and has received honoraria from several such companies in the past, but not in the last three years. PK is listed as inventor on two patents held by University of Birmingham (Atrial Fibrillation Therapy WO 2015140571, Markers for Atrial Fibrillation WO 2016012783). DC receives consulting fees from Bayer, Boehringer Ingleheim, Daiichi-Sankyo and Pfizer. TH is the founder and CEO of Preventicus. The remaining authors declare that the research was conducted in the absence of any commercial or financial relationships that could be construed as a potential conflict of interest.

## Publisher’s Note

All claims expressed in this article are solely those of the authors and do not necessarily represent those of their affiliated organizations, or those of the publisher, the editors and the reviewers. Any product that may be evaluated in this article, or claim that may be made by its manufacturer, is not guaranteed or endorsed by the publisher.

## Footnotes

1
www.preventicus.com

2
www.mmt.ch

## Abbreviations

AA: atrial arrhythmia
AF: atrial fibrillation
ECG: electrocardiogram
PPG: pulse plethysmography.
