# Supplementary material for: Remote Design of a Smartphone and Wearable Detected Atrial Arrhythmia in Older Adults Case Finding Study: Smart in OAC – AFNET 9
Source: Front Cardiovasc Med. 2022 Mar 21;9:839202. doi: 10.3389/fcvm.2022.839202 (PMC8977585; doi:10.3389/fcvm.2022.839202)
Supplement: Supplementary file 1 [file Data_Sheet_1.pdf]

Data supplement. In silico evaluation of the algorithm used to detect atrial arrhythmias using pulse plethysmography data in the Smart in OAC – ANFET 9 study

To quantify which arrhythmias will be classified as atrial arrhythmia by the algorithm used in the Smart in OAC – AFNET 9 study, ECG beat annotations from 7 open databases available from PhysioNet were accessed (see supplementary Table 1) to obtain 1-minute-long beat-to-beat segments. The databases contain data from subjects with: paroxysmal or persistent atrial fibrillation (AF), sinus rhythm, supra-ventricular arrhythmias, atrial, junctional, and ventricular arrhythmias. Overall, from 151838 minutes of rhythm recording from 342 subjects (Supplementary Table 1), 115 minutes and one subject were excluded from the analysis due to being annotated as noise. The rest (151723 minutes from 341 subjects) were analysed using the AF-detection algorithm from Preventicus, replicating the system used in Smart in OAC – AFNET 9. The databases contain either beat and/or rhythm annotations, indicating the type of the beat (normal, premature, ectopic) and/or the rhythm changes. For each one-minute-long segment extracted from the data, all beat types and/or rhythm changes corresponding to it were analyzed by the Preventicus Telecare health system used in the Smart in OAC- AFNET 9 study. To provide an estimate of the arrhythmias classified as atrial arrhythmia by the algorithm used in the study, the rate of atrial fibrillation and atrial flutter were expected to be classified as Smart in OAC “atrial arrhythmia”, supraventricular ectopic rhythms and other atrial irregular rhythms that are neither atrial fibrillation nor atrial flutter were expected to be classified as “normal rhythm with more than 5% irregular beats”, and normal sinus rhythm and non-atrial arrhythmias were expected to be classified as “regular rhythm”.

Results: The algorithm classified 95.8% of the recording showing atrial fibrillation as atrial arrhythmias, and classified 0.3% (sinus rhythm or sinus arrhythmia) to 5.3% (other atrial, junctional, or ventricular arrhythmias) of other recordings showing different rhythms as atrial arrhythmia (Supplementary Table 2). When applying the definition used in Smart in OAC – AFNET 9, accepting only arrhythmias that were 6 minutes or longer at a subject level, the algorithm classified 98% of the subjects with atrial fibrillation or atrial flutter as subjects with AA, and only 1 – 5.6% of subjects with other arrhythmias as subjects with AA. (Supplementary Table 3).

Thus, the algorithm discriminates well between atrial fibrillation and other supraventricular, junctional, or ventricular arrhythmias.

Supplementary Table 1: Detailed description of the databases accessed for the in-silico validation of the algorithm.

Database	Analysable		Description
	subjects	minutes	
Long Term AF Database	79	108279	This database includes **84** long-term ECG recordings of subjects with **paroxysmal** or **sustained atrial fibrillation** (AF). Each record contains two simultaneously recorded ECG signals; record durations vary but are typically **24 to 25 hours.**
St Petersburg INCART 12-lead Arrhythmia Database	75	2250	This database consists of **75 annotated recordings** extracted from **32 Holter** records. Each record is **30 minutes long** and contains 12 standard leads.
MIT-BIH Malignant Ventricular Ectopy Database	22	754	This database includes **22 half-hour ECG** recordings of subjects who experienced **episodes of sustained ventricular tachycardia, ventricular flutter, and ventricular fibrillation.**
MIT-BIH Normal Sinus Rhythm Database	18	23064	This database includes **18 long-term ECG recordings o**f subjects referred to the Arrhythmia Laboratory at Boston's Beth Israel Hospital (now the Beth Israel Deaconess Medical Center). Subjects included in this database were found to have had **no significant arrhythmias**; they include 5 men, aged 26 to 45, and 13 women, aged 20 to 50.
MIT-BIH Atrial Fibrillation Database	23	13742	This database includes **25 long-term ECG recordin**gs of human subjects with **atrial fibrillation (mostly paroxysmal)**. The individual recordings are e**ach 10 hours in duration** and contain two ECG signals.
MIT-BIH Arrhythmia Database	48	1439	The MIT-BIH Arrhythmia Database contains **48 half-hour excerpts** of two-channel ambulatory ECG recordings, obtained from 47 subjects studied by the BIH Arrhythmia Laboratory between 1975 and 1979. Twenty-three recordings were chosen at random from a set of 4000 24-hour ambulatory ECG recordings collected from a mixed population of inpatients (about 60%) and outpatients (about 40%) at Boston's Beth Israel Hospital; the remaining 25 recordings were selected from the same set to include less common but clinically significant arrhythmias that would not be well-represented in a small random sample.
MIT-BIH Supraventricular Arrhythmia Database	77	2310	This database includes **78 half-hour ECG** recordings chosen to supplement the examples of **supraventricular arrhythmias** in the MIT-BIH Arrhythmia Database.
**SUM**	**342**	**151838**	

Supplementary Table 2: Results of the minute-wise analysis in PhysioNet given by rhythm category of each one-minute segment. Each minute was analysed by the algorithm and classified by atrial arrhythmia (AA minute yes) or not (AA minute no).

Minutes*	total	AA minute yes	AA minute no
Atrial Fibrillation	65843	63083 (95.8%)	2760 (4.2%)
Atrial Flutter	91	16 (17.6%)	75 (85.4%)
Supraventricular ectopic beats	9029	234 (2.6%)	8795 (97.4%)
Other supraventricular arrhythmias	111	3 (2.7%)	108 (97.3%)
Non-atrial arrhythmias (junctional or ventricular)	9962	530 (5.3%)	9432 (94.6%)
Sinus rhythm including sinus arrhythmias	66687	181 (0.3%)	66506 (99.7%)

Supplementary Table 3: Results of the subject-wise analysis of presence of atrial arrhythmias lasting ≥6 minutes, given by rhythm category. All minuntes analysed in each patient were combined, and atrial arrhythmias lasting 6 minutes or longer were defined as "AA". This algorithm closely replicates the system used in Smart in OAC during analyzable recordings. The definition "participant with AA" contains 98% of the subjects with atrial fibrillation or atrial flutter. Only a small proportion of subjects with junctional or ventricular arrhythmias (1%) and sinus rhythm including sinus arrhythmias (5.6%) are classified as subjects with AA.

Subjects	total	Subject with AA	Subject without AA
Atrial Fibrillation / atrial Flutter	102	100 (98%)	2 (2%)
Supraventricular ectopic beats	92	0 (0%)	92 (100%)
Other supraventricular arrhythmias	4	0 (0%)	4 (100%)
Non-atrial arrhythmias (junctional or ventricular)	90	1 (1%)	89 (98.9%)
Sinus rhythm including sinus arrhythmias	53	3 (5.6%)	50 (94.3%)

Extraction of pulse signals and differentiation from noise or artefacts.

Noise and artefacts are a key challenge in biosignal analysis. They need to be dealt with when accessing consumer-operated recordings as the signal quality itself has to be taken as it comes. To exclude noise and artefacts from analysis, the detection of atrial arrhythmia in Smart in OAC - AFNET 9 is part of a software and hardware system that includes an artefact identification algorithm which analyses the PPG wave form and detects at pulse-level whether the PPG signal is reliable or not (real signal versus artefact). The artefact identification algorithm analyses several properties of the PPG signal's wave (slope, amplitude, widths) that characterise a pulse wave as real and outputs a score value for each pulse wave. This value is used to classify each signal as real PPG or artefact. If a certain threshold of artefacts is identified in a 1-minute PPG measurement, the recording is excluded from the analysis of atrial arrhythmias.

As a second line of ensuring that artefacts and noise are detected, each recording showing atrial arrhythmias in the study is reviewed by a technician in the preventicus telecare center, including a check for true signals or artefacts. This manual review ensures that only PPG recordings showing the detectable features of atrial arrhythmias are counted and actioned in the study. For further technical details, please refer to Merschel S, Reinhardt L. Analysability of photoplethysmographic smartwatch data by the Preventicus Heartbeats algorithm during everyday life: Feasibility Study. JMIR Form Res (forthcoming). doi:10.2196/29479, accepted Dec 2021.
